# Modeling perinatal mortality in twins via generalized additive mixed models: a comparison of estimation approaches

**DOI:** 10.1186/s12874-019-0861-2

**Published:** 2019-11-15

**Authors:** Muhammad Abu Shadeque Mullah, James A. Hanley, Andrea Benedetti

**Affiliations:** Department of Epidemiology, Biostatistics and Occupational Health, Faculty of Medicine, McGill University, Purvis Hall, 1020 Pine Avenue West, Montreal, QC H3A 1A2 Canada

**Keywords:** Penalized splines, Generalized linear mixed models, Penalized quasi-likelihood, Laplace approximation, Markov chain Monte Carlo, Variance components

## Abstract

**Background:**

The analysis of twin data presents a unique challenge. Second-born twins on average weigh less than first-born twins and have an elevated risk of perinatal mortality. It is not clear whether the risk difference depends on birth order or their relative birth weight. This study evaluates the association between birth order and perinatal mortality by birth order-specific weight difference in twin pregnancies.

**Methods:**

We adopt generalized additive mixed models (GAMMs) which are a flexible version of generalized linear mixed models (GLMMs), to model the association. Estimation of such models for correlated binary data is challenging. We compare both Bayesian and likelihood-based approaches for estimating GAMMs via simulation. We apply the methods to the US matched multiple birth data to evaluate the association between twins’ birth order and perinatal mortality.

**Results:**

Perinatal mortality depends on both birth order and relative birthweight. Simulation results suggest that the Bayesian method with half-Cauchy priors for variance components performs well in estimating all components of the GAMM. The Bayesian results were sensitive to prior specifications.

**Conclusion:**

We adopted a flexible statistical model, GAMM, to precisely estimate the perinatal mortality risk differences between first- and second-born twins whereby birthweight and gestational age are nonparametrically modelled to explicitly adjust for their effects. The risk of perinatal mortality in twins was found to depend on both birth order and relative birthweight. We demonstrated that the Bayesian method estimated the GAMM model components more reliably than the frequentist approaches.

## Background

Twins are 2–4 times more likely to die in the perinatal period compared to singletons [1]. Second-born twins, however, are known to be at higher risk of perinatal mortality than first-born twins [2–4]. While birthweight and gestational age are both well-known determinants of perinatal mortality [5], birthweight is more likely to be a major component of the risk difference between first- and second-born twins because co-twins are usually delivered at the same gestational age. Moreover, second twins, on average, weigh less than first twins [6]. It is unclear if the mortality risk differences between second and first twins depend on birth order or birthweight.

Luo et al. [5] showed that perinatal mortality risk differences in second vs first twins depended on their relative birth size: risks were similar when birthweights were similar, increasingly higher as second twins weighed less, and progressively lower as second twins weighed more. However, in the conditional logistic regression model used, they controlled for the effect of small for gestational age (SGA) via a binary indicator (1 = yes; 0 = no) based on gestational age and birthweight. Controlling for a binary version of continuous confounder(s) may lead to residual confounding [7]. In this analysis, we evaluated the association of birth order with perinatal mortality after adjusting for both birthweight and gestational age, among others.

Because birthweight and gestational age may have nonlinear associations with mortality, we used generalized additive mixed models (GAMMs) [8] that employ unknown smooth functions to model nonlinear covariate effects, and random effects to account for correlation in twin-pairs. Smooth functions can be estimated in various ways [8–10]; here, we used penalized regression splines represented as mixed model components [11]. This allows the use of mixed model methodology and software to make systematic inference on all model components for the GAMMs.

Although several methods are available for estimating GAMMs, in practice results may vary widely depending on the method used. Motivated by the difficulties we encountered when analyzing the perinatal twin mortality, we investigate the performance of different methods via simulation in a setting similar to the twin data situation.

In this paper, we systematically compare the performance of the Bayesian and likelihood-based estimation techniques for inference in the GAMMs via a simulation study. We also apply these methods to the US matched multiple birth data to study the association between birth order and perinatal mortality by birth-order specific weight difference in twins.

## Methods

### Generalized additive mixed models (GAMMs)

Generalized additive mixed models (GAMMs) [8] extend generalized linear mixed models (GLMMs) [12] to allow nonlinear functional forms between independent variables and the response. They provide a flexible modeling framework to use additive nonparametric functions to model the effects of continuous covariate(s) while using random effects to model correlation between responses. Estimating the nonparametric smooth function by penalized regression splines, the GAMM can be expressed as a GLMM. Details on the GAMM and its mixed model representation are provided in the Additional file 1: Supplementary Material.

### Estimation of GAMMs

The GAMM model parameters may be estimated via frequentist or Bayesian approaches. The frequentist approaches rely on approximation methods while Bayesian methods use Markov Chain Monte Carlo (MCMC). We investigate two approximation methods: double penalized quasi-likelihood (DPQL) [8, 13] and the Laplace approximation [14], as well as a Bayesian approach [15].

The Bayesian methods require specification of prior distributions, a non-trivial task for variance components [16]. For the GAMM, an appropriate choice for the prior distributions of variance components is crucial because curve estimation depends on the variance components; over (under) estimation of the variance components corresponds to undersmoothing (oversmoothing).

## Analysis of twins perinatal mortality data

### Methods

#### Data and models

To study the relationship between perinatal mortality and birth order, we used the matched multiple birth dataset from the United States National Centre for Health Statistic’s (NCHS) 1995–1998. For all multiple births in years 1995–1998, the NCHS data contained information on perinatal and infant mortality, and maternal and pregnancy characteristics. An extended version (1995–2000) of this dataset was used by Luo et al. [5].

There were a total of 446,570 matched births. We excluded (15.6% of the total) matched births with the following criteria due to missing or implausible data: (i) triplet and higher order multiple birth (*n* = 23,672); (ii) unknown breech presentation (*n* = 5041); (iii) unmatched twins (*n* = 3650; see, Martin et al. [17] for details); (iv) twins with unknown birth order (*n* = 3507); (v) extreme gestational ages (< 23 weeks or > 42 weeks, *n* = 17,475); (vi) extreme birthweights (< 500 g or > 6000 g, *n* = 4589); (vii) twins not delivered at the same gestational week (*n* = 9918); and (viii) birthweight difference between second and first twins greater than 100% (*n* = 1758). The final study cohort included 376,960 twin births in 188,480 twin pregnancies.

To assess perinatal mortality risk differences between second- and firstborn twins by birth order-specific weight difference, we conducted a stratified analysis following Luo et al. [5]. Based on the birthweight difference between twins, we divided the dataset into 7 strata as follows: (i) within ±5% (similar); (ii) first twins heavier by 5–15%; (iii) first twins heavier by 15–25%; (iv) first twins heavier by ≥ 25%; (v) second twins heavier by 5–15%; (vi) second twins heavier by 15–25%; and (vii) second twins heavier by ≥ 25%.

In order to estimate the odds ratio (OR) and 95% confidence interval (CIs) of perinatal death comparing second vs first twins in each stratum, we used GAMMs. This approach is broadly similar to the conditional logistic regression approach of Luo et al. [5]. We adjusted for potential confounders including fetal sex, presentation, birthweight, gestational age, and mode of delivery. Birthweight and gestational age effects were modelled nonparametrically. We did not adjust for maternal characteristics or any other factors common to a twin pair as these were perfectly matched for twins.

More specifically, conditional on twin-pair-specific random intercepts *b*_*hi*_ *~ N(0, σ*^*2*^_*int*_*),* the binary outcomes *Y*_*hij*_ (perinatal death: 0 = no, 1 = yes) in *h*^th^ stratum *(h = 1, …, 7)* were assumed to be independent and follow a semiparametric logistic mixed model

1 $$ \mathrm{logit}\left\{\Pr \left({Y}_{hi j}=1|{b}_{hi}\right)\right\}={x}_{hi j}^T\beta +{f}_1\left({\mathrm{birthweight}}_{hi j}\right)+{f}_2\left(\mathrm{gestational}\ {\mathrm{age}}_{hi j}\right)+{b}_{hi}, $$

where *i = 1, ..., m*_*h*_ indexes the twin pair in *h*^th^ stratum and *j = 1, 2* twins within pairs, the fixed effects covariates *x*_*hij*_ included an intercept, birth order, fetal sex, presentation, and mode of delivery; *f*_*1 *_(birthweight_*hij*_) and *f*_*2 *_(gestational age_*hij*_) are centred twice-differentiable smooth functions of birthweight and gestational age, respectively. The random intercept variance and all other model parameters are stratum-specific and *m*_*h*_ denotes the number of twin-pairs in the *h*^th^ stratum. Note that, in our analysis, birthweight and gestational age were linearly correlated *(r = 0.72)*. The correlations between all other covariates were negligible.

#### Data analysis

We fitted model (1) in each stratum in which each smooth term was represented by a penalized thin plate regression spline [18, 19]. Following Ruppert [20], we considered a large number of knots (*K =* 20) and knot positions were evenly spaced sample quantiles of unique covariate values. Representing the penalized regression smoothers as mixed model components, and imposing the centering constraint on each smoother, we estimated the model parameters via the following methods:

1. DPQL under maximum likelihood (ML) estimation.
2. Laplace approximation. For DPQL and Laplace methods, standard errors of the estimated fixed effects and smooth functions were obtained from a posterior covariance matrix as in Lin and Zhang [8].
3. A Bayesian approach in which noninformative priors were used for all parameters. Specifically, *N*(0, 10^6^) distributions were used for all fixed effects (*β*), while half-Cauchy priors [16] with scale parameter set to 25 were considered for each variance component (e.g., for *σ*^*2*^_*int*_). We ran 2 chains with 55,000 iterations after discarding the initial 5000 burn-in iterations. The chains were thinned by keeping every 50th iteration and estimates were the sample medians. Convergence of the chains was assessed following Gelman and Rubin [21] and also by visually examining the trace plot, density plot, and sample autocorrelation function for each parameter.

All analyses were carried out in R software employing glmmPQL [22] and gamm4 [23] functions for DPQL and Laplace approximate methods, respectively. The Bayesian analysis via MCMC was performed using JAGS [24] which is a mature and declarative language for Bayesian model fitting.

### Results of the data analysis

Table 1 presents the summaries of selected characteristics of the twins study cohort. Most mothers were white (79.3%) and aged between 20 and 34 (74.8%). Second-born twins had slightly lower mean birthweights (23.6 g) than first-born twins. Malpresentation was more frequent in second twins (27% vs 21%).

**Table 1:** Characteristics of mothers and twin births included in the twins perinatal mortality study

Characteristic	Mothers	Twins	
		First Born	Second Born
Mothers, n (%)	188480		
Race			
White	149459 (79.3)		
Black	31912 (16.9)		
Other	7109 (3.8)		
Age			
< 20	13192 (7.0)		
20–34	140992 (74.8)		
≥ 35	34296 (18.2)		
Newborns^a^		188480	188480
Sex, boy		94326 (50.1)	94654 (50.2)
Gestational age, week		35.7 (3.2)	35.7 (3.2)
Birth weight, gram		2407.5 (615.5)	2383.9 (618.5)
Breech/Malpresentation		40832 (21.7)	51661 (27.4)
Cesarean		100271 (53.2)	108413 (57.5)

^a^We report mean (SD) for quantitative variables, and count (percentage) for categorical variables

The ORs of perinatal death in second versus first twins from the models fit via Laplace and Bayesian-HC are reported in Table 2. DPQL estimation failed in all strata and did not converge even for simpler models, e.g., when the effects of birthweight and gestational age were considered as linear, or, when some confounders were dropped. The Laplace method yielded more extreme ORs (away from 1) with wider CIs than the Bayesian method for all strata except for one where they were approximately equal (when second-born twins were heavier by 5 to 15%). From the Bayesian fitting, the risk of perinatal death was higher for second-born twins (adjusted OR 1.27, 95% CI: 1.13, 1.43) when the twins had similar birthweights (within ±5%) and the risk increased as the second-born twin weighed less. When second twins were heavier by 5–15% and 15–25%, the adjusted ORs were not significantly different from 1. Second-born twins were found to be at significantly lower risk of perinatal death (adjusted OR 0.33, 95% CI: 0.25, 0.45) when they weighed ≥ 25% more than the first-born twin.

**Table 2:** Stratified comparisons of second and firstborn twins: rates and ORs of perinatal death

Variable	Twin births n(%)	Perinatal death n (per 1000)		OR^a^ (95% CI)		Variance of random intercepts	
		Firstborn	Secondborn	Laplace Fit	Bayesian Fit^b^	Laplace Fit	Bayesian Fit
Birth weight, heavier in %^c^							
Heavier firstborn twin							
≥ 25%	32,940 (8.74)	358 (21.74)	989 (60.05)	4.15 (2.31, 6.13)	3.42 (2.47, 4.70)	104.2	3.5
15 to < 25%	35,810 (9.50)	295 (16.48)	490 (27.37)	2.31 (1.46, 3.65)	1.97 (1.58, 2.49)	74.7	5.5
5 to < 15%	72,230 (19.16)	565 (15.64)	723 (20.02)	1.68 (1.33, 2.12)	1.39 (1.20, 1.62)	42.3	4.4
Similar birth weight							
within ±5%	109,998 (29.18)	1040 (18.91)	1174 (21.35)	1.48 (1.28, 1.72)	1.27 (1.13, 1.43)	31.8	5.4
Heavier secondborn twin							
5 to < 15%	69,804 (18.52)	617 (17.68)	608 (17.42)	1.16 (0.90, 1.50)	1.19 (0.97, 1.40)	69.9	4.7
15 to < 25%	32,118 (8.52)	354 (22.04)	334 (20.80)	0.86 (0.67, 1.12)	0.91 (0.71, 1.20)	73.9	5.6
≥ 25%	24,060 (6.38)	506 (42.06)	351 (29.18)	0.14 (0.07, 0.26)	0.33 (0.25, 0.45)	107.5	3.3

^a^Adjusted ORs comparing second vs first twin from logistic additive mixed effects models adjusting for fetal sex, birth weight, gestational age, presentation (breech/malpresentation: YES/NO), and mode of delivery (cesarean: YES/NO). Given the death rate is very low, the ORs are good approximation of rate ratios (RRs)

^b^Bayesian estimation with half-Cauchy prior for the variance component (Bayesian-HC method)

^c^Birthweight difference in percentage comparing the heavier vs lighter twins

The variance estimates of the random intercepts are shown in the last two columns of Table 2. Estimates obtained from the Laplace method were implausibly large (up to thirty times bigger than those of the Bayesian method), possibly because the Laplace approximation performs poorly for binary data with small cluster size (*n*_*i*_ = 2) and very low event probability, or, because the method did not converge well without reporting any warning. The Bayesian estimates of the variance of random effects indicated large heterogeneity between twin pairs (nearly 5 in most strata).

**Fig. 1:**
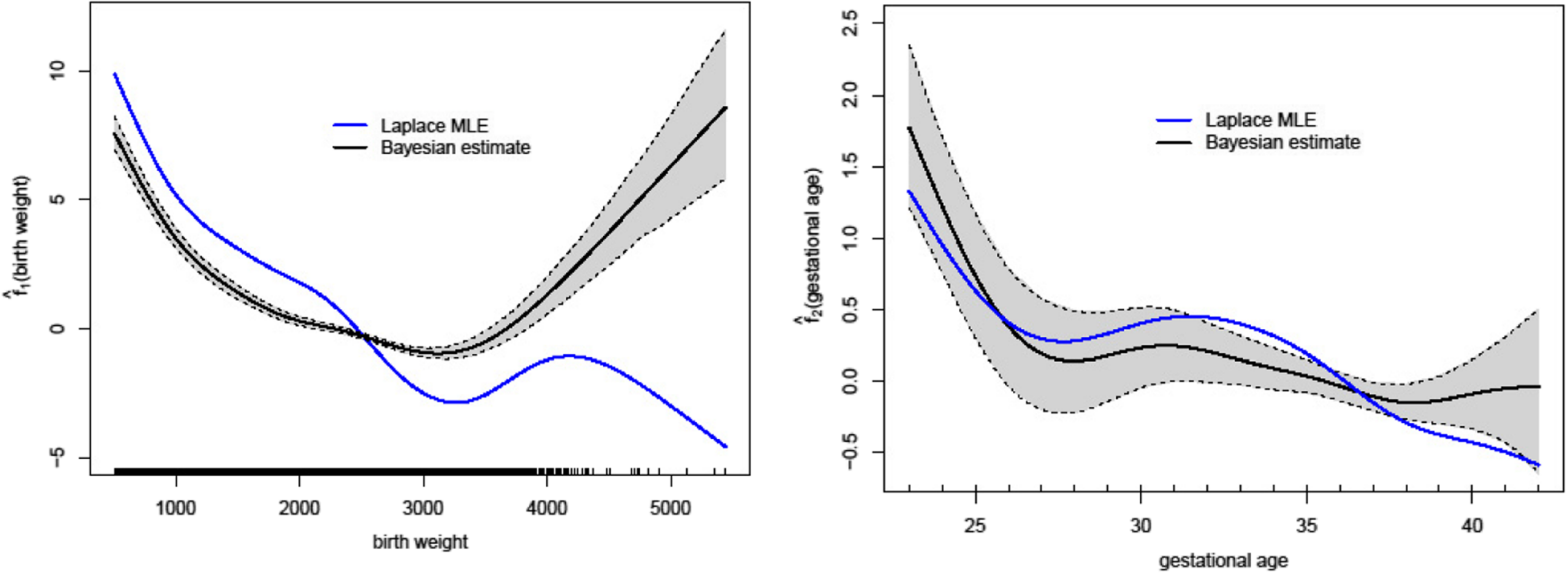
Bayesian and Laplace estimates of *f*_*1 *_(birthweight) at the left panel, and *f*_*2 *_(gestational age) at the right panel for the twins mortality data. The shaded regions are the pointwise 95% credible sets obtained from the fully Bayesian fit

Figure 1 shows the estimated nonparametric functions of birthweight and gestational age for the Laplace and Bayesian-HC methods when birthweights for first- and second-born twins were similar. The 95% pointwise credible intervals of the curves are displayed only for the Bayesian method. The estimated curves suggested different trends especially for birthweight. The Bayesian-estimated curve indicated an increased risk of perinatal mortality at extreme birth weights (< 1000 g and > 5000 g). The Laplacian fit showed a decreasing trend for (mortality) risk as birthweight increased. The risk of perinatal death estimated by the Bayesian method declined sharply up until 28 weeks of gestational age and declined gradually thereafter although there was a slight increase in risk after 40 weeks. The Laplacian fit did not closely reproduce the behaviour of the Bayesian fit. The fitted curves obtained from other strata were similar to those shown in Fig. 1.

In summary, the estimated ORs by different methods disagreed by a noticeable margin; the shape of the nonlinear associations varied widely, one method failed to converge, and the variance component estimates differed markedly. Because it was unclear which estimates should be reported, we conducted a simulation study to investigate the performance of the Bayesian and frequentist approaches for estimating GAMMs under twin data setting.

## Simulation study

### Methods

#### Data generation: mimicking twin-pairs data setting

The simulation study was broadly designed to mimic the twin-pairs data setting where the cluster size was 2. For all settings, data were generated following Lin and Zhang [8]. Each dataset was generated with *m* = 1000 clusters of homogeneous size *n*_*i*_ = 2. We considered a random intercepts model with four variables: a binary outcome (Y), a dichotomous variable (*D*) such as birth order, and two continuous covariates (*X*_*1 *_*, X*_*2*_) such as birthweight and gestational age. The dichotomous variable, *D* was generated from a Bernoulli distribution with probability equal to 50%. Two linearly correlated standard uniform *U*(0, 1) covariates (*X*_*1*_ and *X*_*2*_) were generated from the Gaussian Copula such that the point-biserial correlations between the dichotomous variable and continuous covariates were *ρ*_*d.x1*_ = *ρ*_*d.x2*_ ≈ 0.30. The empirical correlation between the continuous covariates was considered as *ρ*_*x1. x2*_ = 0.7, which is similar to the observed correlation between birthweight and gestational age observed in the twin-pairs data. Conditional on the cluster-specific random intercepts *b*_*i*_ ~ *N*(0, 0.75), the binary responses *Y*_*ij*_ within each cluster were generated with conditional probabilities:

2 $$ \mathrm{logit}\ \left({p}_{ij}\right)={\beta}_0+{\beta}_1D+{f}_1\left({x}_{1 ij}\right)+{f}_2\left({x}_{2 ij}\right)+{b}_i, $$

where ${p}_{ij}=\mathbbm{E}\left[{Y}_{ij}|{b}_i\right]$, *β*_1_ = 0.7 (which gives OR ≈ 2)

3 $$ {f}_1\left({x}_1\right)=\frac{1}{20}\left\{\frac{6\Gamma (37)}{\Gamma (30)\Gamma (17)}{x}_1^{29}{\left(1-{x}_1\right)}^{16}+\frac{4\Gamma (14)}{\Gamma (3)\Gamma (11)}{x}_1^2{\left(1-{x}_1\right)}^{10}\right\}-\frac{1}{2}, $$

4 $$ {f}_2\left({x}_2\right)=\frac{1}{6}\left\{\frac{2\Gamma (16)}{\Gamma (8)\Gamma (8)}{x}_2^7{\left(1-{x}_2\right)}^7+\frac{\Gamma (10)}{\Gamma (5)\Gamma (5)}{x}_2^4{\left(1-{x}_2\right)}^4\right\}-\frac{1}{2}, $$

and Γ(.) is a gamma function. The overall prevalence of a positive (*Y =* 1) outcome was kept at either 0.05 or 0.5 by setting *β*_*0*_ to either − 1.28 or − 0.35. Here *f*_*1 *_*(x*_*1*_*)* and *f*_*2 *_*(x*_*2*_*)* were bimodal and unimodal functions, respectively. We centered the functions so that the means of *f*_*1*_ and *f*_*2*_ were 0 over the distinct values of *X*_*1*_ and *X*_*2*_.

#### Analysis of the simulated data

For each setting, 1000 simulated datasets were generated. Each dataset was analysed by fitting a logistic additive mixed effects model of the form (2) in which each smooth term was represented by penalized thin plate regression spline similar to twin data analysis. The model parameters were estimated using the following methods:

1. DPQL under maximum likelihood (ML) or restricted ML (REML) estimation.
2. Laplace approximation. For DPQL and Laplace methods, standard errors of the estimated fixed effects and smooth functions were obtained following the same procedure as for the twin data analysis.
3. A Bayesian approach similar to those used for perinatal mortality data analysis with *β* ~ *N*(0, 10^6^) but considering three alternative independent prior specifications for each variance component: (i) Uniform (0, 100); (ii) Half-Cauchy with scale parameter set to 25; and (iii) Inverse Gamma (0.001, 0.001). Using priors (i)-(iii), Bayesian methods are referred to later as, respectively: Bayesian-UNIF, Bayesian-HC, and Bayesian-IG. The Bayesian estimates were medians from 55,000 iterations of the MCMC algorithm after discarding the first 5000 iterations as burn-in. We ran a single chain and thinned it by keeping every 50th iteration.

#### Performance indicators

We computed percentage relative biases (PRBs) of the fixed and random effects estimators defined as

$$ \mathrm{PRB}=\frac{\mathrm{Bias}}{\mathrm{True}\ \mathrm{Value}}\times 100. $$

For the estimated smooth functions, we computed pointwise mean average squared distance/error (MASE) from the true curves, the 95% pointwise mean average coverage probabilities (MACPs), and 95% pointwise mean average confidence interval lengths (MACLs). The pointwise MASE was defined as the mean over the 1000 replicated datasets of the average squared error,

$$ \mathrm{ASE}={\left(\sum \limits_{i=1}^m{n}_i\right)}^{-1}\sum \limits_{i=1}^m\sum \limits_{j=1}^{n_i}{\left\{\hat{f}\left({x}_{lij}\right)-f\left({x}_{lij}\right)\right\}}^2;l=1,2. $$

The 95% pointwise MACP and MACL were obtained as the means of the 1000 average coverage probabilities (ACP) and average credible intervals lengths (ACL), respectively. We defined

$$ \mathrm{ACP}={\left(\sum \limits_{i=1}^m{n}_i\right)}^{-1}\sum \limits_{i=1}^m\sum \limits_{j=1}^{n_i}\mathbbm{1}\left({\hat{f}}_L\left({x}_{lij}\right)<f\left({x}_{lij}\right)<{\hat{f}}_U\left({x}_{lij}\right)\right), $$

$$ \mathrm{ACL}={\left(\sum \limits_{i=1}^m{n}_i\right)}^{-1}\sum \limits_{i=1}^m\sum \limits_{j=1}^{n_i}\left({\hat{f}}_U\left({x}_{lij}\right)-{\hat{f}}_L\left({x}_{lij}\right)\right), $$

where $\mathbbm{1}$(.) denotes an indicator function; ${\hat{f}}_L$ and ${\hat{f}}_U$ are the lower and upper limits of the point-wise CI, respectively.

### Results of the simulation study

Table 3 presents simulation results when different methods of estimating GAMMs were used. For low (5%) event probability setting, model components were better estimated by Bayesian methods than the two frequentist approaches with lower percent relative bias and MSE, shorter distance between the true and estimated curves, and higher coverage probability. Of the Bayesian methods, Bayesian-HC performed best in estimating most of the model components. Both DPQL (ML and REML) and Laplace methods yielded inflated ${\hat{\sigma}}_{int}^2$ and showed poor ability in recapturing the true functions. DPQL (ML and REML) failed to converge often (66% of total datasets) whereas the Laplace method had negligible convergence problems (2% of total datasets), and results from these datasets were excluded. The Bayesian methods did not report any convergence problems. The Bayesian-UNIF method overestimated ${\sigma}_{int}^2$ whereas Bayesian-IG underestimated it. The fixed effect *β*_*1*_ was better estimated by Bayesian methods with negligible bias and lower MSE. The Bayesian-HC appeared to perform in between the Bayesian-UNIF and Bayesian-IG methods in terms of estimating all model components. Overall, the Bayesian estimates were sensitive to the prior specifications of the variance component. The simulation results suggest that the Bayesian-HC is likely to be the best approach in the twins-pair data, where the event rate of interest is < 5%. Increasing the event rate from 5 to 50% resulted in better estimates by all methods without any convergence problems. The superiority of the Bayesian methods however prevailed.

**Table 3:** Estimate, 95% confidence/credible interval (CI), mean average squared distance (MASE), mean average 95% coverage probability (MACP), and mean average coverage length (MACL) for model parameters estimated via various approaches when number of clusters *m* = 1000, cluster size *n*_*i*_ = 2, and ${\rho}_{x_1.{x}_2}=0.7$

Method	${\sigma}_{int}^2=0.75$			*β*_*trt*_ = 0.7			*f*_1_(*x*_1_)			*f*_2_(*x*_2_)		
	${\hat{\sigma}}_{int}^2$	PRB	95% CI	${\hat{\beta}}_{trt}$	PRB	95% CI	MASE	MACP	MACL	MASE	MACP	MACL
Event probability = 0.05												
DPQL (ML)	15.83	2010.60	(4.56, 27.10)	1.24	76.44	(0.23, 2.24)	7.510	0.32	1.71	11.784	0.33	1.72
DPQL (REML)	30.45	3959.66	(16.39, 44.56)	1.07	52.38	(0.01, 2.13)	6.472	0.34	1.79	12.584	0.34	1.70
Laplace ML	56.02	7369.66	(5.71, 106.34)	0.79	13.21	(0.07, 1.51)	0.763	0.70	1.76	0.907	0.73	1.82
Bayesian (Uniform Prior)	0.96	27.42	(0.06, 2.88)	0.75	6.54	(0.28, 1.25)	0.148	0.94	1.39	0.112	0.94	1.24
Bayesian (Half-Cauchy Prior)	0.87	15.40	(0.06, 2.71)	0.72	3.25	(0.29, 1.22)	0.142	0.94	1.27	0.103	0.94	1.15
Bayesian (IG Prior)	0.39	−48.40	(0.01, 2.20)	0.71	1.71	(0.27, 1.18)	0.149	0.93	1.25	0.103	0.93	1.13
Event Probability = 0.5												
DPQL (ML)	0.87	15.70	(0.40, 1.34)	0.64	−8.55	(0.42, 0.86)	0.032	0.89	0.57	0.023	0.88	0.48
DPQL (REML)	0.98	30.92	(0.52, 1.44)	0.66	−5.86	(0.42, 0.90)	0.028	0.91	0.58	0.024	0.90	0.47
Laplace ML	0.36	−52.49	(0.12, 0.60)	0.66	−5.77	(0.43, 0.89)	0.037	0.87	0.58	0.024	0.87	0.49
Bayesian (Uniform Prior)	0.82	8.99	(0.33, 1.40)	0.71	1.35	(0.47, 0.97)	0.032	0.95	0.70	0.024	0.95	0.61
Bayesian (Half-Cauchy Prior)	0.80	6.04	(0.34, 1.36)	0.71	0.76	(0.47, 0.96)	0.032	0.95	0.67	0.023	0.95	0.58
Bayesian (IG Prior)	0.72	−6.50	(0.19, 1.30)	0.69	−0.92	(0.47, 0.95)	0.033	0.94	0.68	0.023	0.95	0.58

For the Bayesian method, the three alternative priors used for the variance components were: uniform (0, 100), half-Cauchy (25), and inverse gamma, IG(0.001, 0.001)

**Fig. 2:**
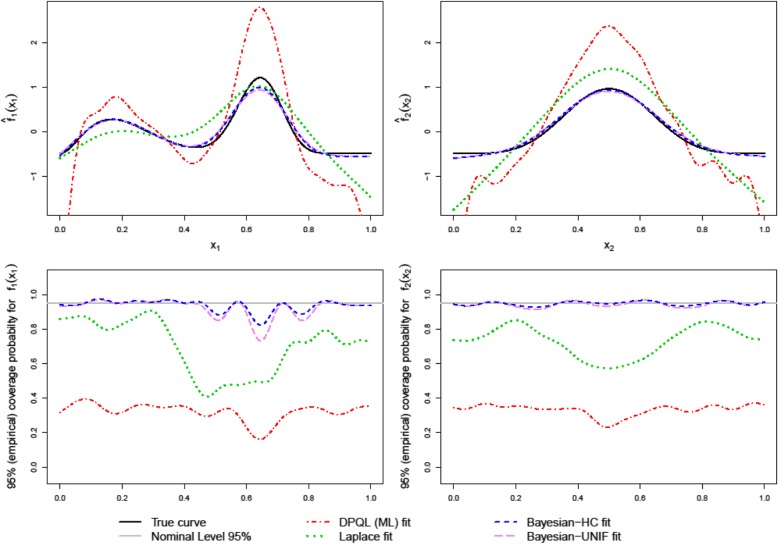
True and estimated curves of the estimated nonparametric functions based on 1000 replications (${\hat{f}}_1\left({x}_1\right)$ and ${\hat{f}}_2\left({x}_2\right)$ in the upper panel) and smoothed pointwise coverage probabilities of the 95% confidence intervals (*f*_*1 *_*(x*_*1*_*)* and *f*_*2 *_*(x*_*2*_*)* in the lower panel) for 1000 replicated datasets. These results are for the data generation scenario with *m* = 1000, *n*_*i*_ = 2, ${\sigma}_{int}^2$ = 0.75, ${\rho}_{x_1.{x}_2}$ = 0.7 and event probability = 0.05. The curves estimated by Bayesian-IG were almost similar to those obtained by Bayesian-HC and have not been displayed here to make other fits more visible

Figure 2 illustrates the ability of the GAMMs estimated by different methods to recapture the true functions when the event probability was 0.05. The upper panel of Fig. 2 shows the true curves *f*_*j*_ *(x*_*j*_*) (j = 1, 2)* and the smoothed estimated curves ${\hat{f}}_j\left({x}_j\right)$. The Bayesian methods recovered the true curves well with slightly negative biases when curvature was high and also in the flat areas. The fitted curves obtained by Bayesian methods using three different priors for variance components were similar. However, around the peak area the Bayesian-HC method performed better than others. Bayesian-UNIF and Bayesian-IG yielded almost identical curves and hence the curves obtained by Bayesian-IG have not been displayed. The frequentist methods, in contrast, could not adequately recapture the true curves throughout the range. DPQL (ML) performed worse than the Laplace approximation method.

The lower panel of Fig. 2 compares the empirical pointwise coverage probabilities of the 95% credible intervals of *f*_*1 *_*(x*_*1*_*)* and *f*_*2 *_*(x*_*2*_*)* obtained using different estimation methods. The coverage probabilities (CP) of the CIs from all Bayesian methods were close to the nominal value (95%), except when biases in the estimated nonparametric functions were noticeable. In contrast, CIs from DPQL (ML) and Laplace methods yielded very low coverage probabilities, and DPQL (ML) had relatively poor coverage probabilities (mean CP 33%) compared to the Laplace method (mean 73%). Around the peak areas, CIs from DPQL (ML) and Laplace methods yielded very low coverage probabilities. In such cases, coverage probabilities from the fully Bayesian methods were also low, but nonetheless much better than frequentist methods.

## Discussion

We re-analyzed twins perinatal mortality data to study the association between birth order and perinatal mortality by adopting the flexible GAMMs in which continuous covariates (birthweight and gestational age) were nonparametrically modelled to adjust for their effects more completely. Overall, how best to estimate flexible regression curves when the outcomes are correlated and binary is unclear, especially when cluster sizes are small. Thus, we analyzed twins data estimating GAMMs by different frequentist and Bayesian methods, and used simulated data to compare the performance of these estimation techniques for a setting similar to the twin-data.

Using the multiple matched data from the US National Centre for Health Statistic’s (NCHS) 1995–1998, we obtained results that varied with respect to the estimation methods. Our simulation results for small cluster size (*n*_*i*_ = 2) with low event probability (similar to the NCHS data) suggested the superiority of the Bayesian method in estimating all model components, especially using the Half-Cauchy (HC) priors for the variance components. We thus rely on the results from the Bayesian-HC fit for our data analysis. These results suggest that the risk of perinatal mortality depended on the twins’ birth order and the risk differences in second vs first twins depended on their relative birthweight. Second twins were more likely to die than first-born co-twins when they had similar (within ±5%) birthweights (adjusted OR = 1.27, 95% CI: 1.13, 1.43). The risks of perinatal death for second-born twins were progressively higher as they weighed less than first-born twins (adjusted ORs: 1.39, 1.97 and 3.42 when weighed 5–15%, 15–25% and ≥ 25% less, respectively) and increasingly lower as they weighed more (adjusted ORs: 1.19, 0.91 and 0.33 when weighed 5–15%, 15–25% and ≥ 25% more, respectively; most of the ORs were significantly different from 1). Similar to the simulation results, the Bayesian analysis using uniform priors for variance components (Bayesian-UNIF) yielded slightly larger ORs whereas using inverse gamma priors (Bayesian-IG) yielded slightly smaller ORs as compared to the Bayesian-HC method (see Additional file 1: Table S1 for the results).

The effect of relative birthweight was also confirmed by Luo et al. [5] but they did not find any significant association between birth order and perinatal mortality when both twins had similar (within ±5%) birthweights (OR = 0.97, 95% CI: 0.84, 1.12). Also, the ORs they obtained from the stratified analyses were closer to 1 in most cases. This may be due to using different models, or adjusting for different sets of confounders. They used a binary indicator ‘small for gestational age’ to control for the effect of birthweight and gestational age, which might lead to residual confounding [7].

Similar to the findings from the simulation study, the Laplace estimate of the variance of the random intercepts in each stratum was unusually large - indicating an extreme heterogeneity between twin pairs. The fitted smooth curves for birthweight and gestational age by the Laplace method were less likely to capture the true shapes of association due to the poor estimates of the variance components. The curve estimation largely depends on the estimates of the variance components in a GAMM, and the Laplace method yielded poor estimates of the variance components as evident from the simulation study. The DPQL method failed to fit the model in each stratum and this was in agreement with the findings from the simulation study in which DPQL failed to converge often.

The observed performance of the DPQL and Laplace approximation in estimating the model components in the simulation study was not surprising as they are known to yield biased estimates for small cluster size. However, we demonstrated the strength of Bayesian methods when the system was stressed, i.e., when cluster size and event probability were small. While using Frequentist methods with more refined likelihood approximation (e.g. adaptive Gaussian quadrature) may improve performance but is not feasible as the mixed model representation of GAMMs involves a large number of random effects and Gaussian quadrature is not computationally efficient for more than four random effects [25].

There are some limitations to this study. First, we analyzed the NCHS 1995–1998 twin matched data that we had access to. Unfortunately, the updated version of the data from 1995 to 2000 was not publicly available during this analysis. We do not believe that the results would have changed appreciably given two more years of data. Next, we considered a stratified analysis for twin-data analysis, although a single model that included an interaction term for birth order and relative birth size might be more appropriate to study their association with perinatal mortality by estimating these effects using the whole dataset at once. Stratification was used to make our results comparable to Luo et al. [5], and to reduce the computational resources required for handling a huge dataset. Finally, we omitted a potential confounder, zygosity (Monozygotic-MZ/Dizygotic-DZ), because the data was not available. MZ twins are likely to be more correlated both for birthweight and for potential mortality than DZ twins.

## Conclusion

We adopted a sophisticated statistical model, GAMM, to precisely estimate the perinatal mortality risk differences between first- and second-born twins from a large dataset in which birthweight and gestational age were nonparametrically modelled to explicitly adjust for their effects. Overall, the perinatal mortality risk differences in second vs first twins were found to depend on both birth order and relative birthweight. We demonstrated that the Bayesian method (especially using half-Cauchy prior for variance component) estimates the GAMM model components more reliably than the frequentist approaches for small cluster size.

## Supplementary information

**Additional file 1: **Supplementary Material: The Mixed Model Representation of the GAMMs. This file contains an explanation on the generalized additive mixed models (GAMMs) and their representation as generalized linear mixed models (GLMMs). In addition, it includes a table that summarizes additional results from the twin perinatal mortality data analysis. **Table S1.** Stratified comparisons of second and firstborn twins: adjusted ORs of perinatal death obtain from the Bayesian fit of the logistic additive mixed effects models.

## Data Availability

In this paper we use secondary data on Matched Multiple Birth from the United States National Centre for Health Statistic’s (NCHS) Vital Statistics 1995–1998. The data are available online (https://www.nber.org/data/vital-statistics-matched-multiple-births-data.html).

## Abbreviations

DPQL: Double penalized quasi-likelihood
GAMM: Generalized additive mixed models
GLMM: Generalized linear mixed model
MACP: Mean average coverage probability
MASE: Mean average squared error
MCMC: Markov chain Monte Carlo
REML: Restricted maximum likelihood
SGA: Small for gestational age
