# Supplementary material for: Modeling perinatal mortality in twins via generalized additive mixed models: a comparison of estimation approaches
Source: BMC Med Res Methodol. 2019 Nov 15;19:209. doi: 10.1186/s12874-019-0861-2 (PMC6858726; doi:10.1186/s12874-019-0861-2)
Supplement: Supplementary file 1 — Additional file 1: Supplementary Material: The Mixed Model Representation of the GAMMs. This file contains an explanation on the generalized additive mixed models (GAMMs) and their representation as generalized linear mixed models (GLMMs). In addition, it includes a table that summarizes additional results from the twin perinatal mortality data analysis. Table S1. Stratified comparisons of second and firstborn twins: adjusted ORs of perinatal death obtain from the Bayesian fit of the logistic additive mixed effects models. [file 12874_2019_861_MOESM1_ESM.pdf]

Modeling perinatal mortality in twins via generalized additive mixed models: a comparison of estimation approaches

Muhammad Abu Shadeque Mullah[1], James A Hanley[1] and Andrea Benedetti*[1]

[1]Department of Epidemiology, Biostatistics and Ocuupational Health, McGill University

Supplemetary Material

*Correspondence to Dr. Andrea Benedetti, Department of Epidemiology, Biostatistics, and Occupational Health, Faculty of Medicine, McGill University, Purvis Hall, 1020 Pine Avenue West, Montreal, QC H4B 2V2, Canada (e-mail: andrea.benedetti@mcgill.ca).

The Mixed Model Representation of the GAMMs

Generalized Additive Mixed Models (GAMMs)

Suppose the observations for the jth member of the ith cluster consist of a response variable Y_{ij} ($i = 1, 2, \ldots, m; j = 1, 2, \ldots, n_i$) and p covariates $x_{ij} = (x_{1ij}, \ldots, x_{pij})^T$ associated with fixed effects and $q \times 1$ vector of covariates z_{ij} associated with random effects. For the twin-pairs data, each twin-pair is considered as a cluster so that the cluster size $n_i = 2$ for all clusters, and m is the total number of twin-pairs. Let $Y_i = (Y_{i1}, \ldots Y_{in_i})^T$ and define x_i and z_i similarly. Given the $q \times 1$ vector of random effects, b, the generalized additive mixed models takes the form

$$g(\mu_i) = \beta_0 + f_1(x_{1i}) + \cdots + f_p(x_{pi}) + z_i^T b, \quad (1)$$

where $\mu_i = E(Y_i \mid b)$, $g(\cdot)$ is a strictly monotone and differentiable link function, $f_r(\cdot)(r = 1, \ldots, p)$ is a centered and twice-differentiable smooth function, and the random effects b are assumed to be distributed as $N(0, D)$.

Penalized Spline Estimation

We model the smooth function $f_r(\cdot)$ by using the thin plate regression splines of Wood [19]. Taking a large number of knots (K_r) to ensure the desired flexibility, we form a basis matrix B_r whose elements are thin plate regression spline basis functions $B_{rk}(x_{ri})$. We then represent $f_r(\cdot)$ by a regression spline

$$f_r = B_r \beta_r \quad (2)$$

and prevent overfitting by imposing the restriction

$$\beta_r^T S_r \beta_r \leq c \quad (3)$$

for some nonnegative constant c, where $\beta_r = (\beta_{r1}, \ldots, \beta_{rKr})^T$ is a vector of basis coefficients, and S_r is a positive semi-definite penalty matrix. To ensure identifiability we choose B_r and β_r such that

$$\mathbf{1}^T B_r \beta_r = 0, \quad (4)$$

where $\mathbf{1}$ is a vector of all 1s.

The Penalized Spline as a Mixed Model

Following Wood [25], we re-parameterize $f_r(\cdot)$ in terms of a fixed effects parameter vector β_{rF} and a random effects β_{rR} via a one-to-one transformation as

$$f_r = B_{rF}\beta_{rF} + B_{rR}\beta_{rR}, \quad (5)$$

where $\beta_{rR} \sim N(0, \tau_r I)$ with $\tau_r = 1/\lambda_r$, B_{rF} are the columns of B_r for which the penalty matrix S_r has zero eigenvalues, and $B_{rR} = B_r U_r \sqrt{D_r^{-1}}$ in which U_r is the matrix containing eigenvectors of S_r corresponding to the strictly positive eigenvalues arranged in descending order of magnitude (E_r), and D_r is the diagonal matrix containing E_r on the leading diagonal.

GAMMs as Generalized Linear Mixed Models (GLMMs)

Substituting (5) in (1), we obtain

$$g(\mu) = B_F\beta_F + B_R\beta_R + Zb, \tag{6}$$

where $\mu = (\mu_1, \ldots, \mu_m)^T$, $B_F = (\mathbf{1}, B_{1F}, \ldots, B_{pF})$ is the fixed effects design matrix, $B_R = (B_{1R}, \ldots, B_{pR})$ is the random effects design matrix associated with the smooth functions, $Z = (z_1, \ldots, z_q)$ is the typical random effects model matrix, $\beta_F = (\beta_0, \beta_{1F}^T, \ldots, \beta_{pF}^T)^T$ is a vector of fixed effect parameters, and $\beta_R = (\beta_{1R}^T, \ldots, \beta_{pR}^T)^T$ and b are independent random effects with distributions $\beta_R \sim N(0, \Lambda_\tau)$ and $b \sim N(0, D_\gamma)$ in which $\Lambda_\tau = \text{diag}\,(\tau_1 I, \ldots, \tau_p I)$. The GAMM (6) can be written more compactly in the form of a GLMM as

$$g(\mu) = \underline{X}\beta_F + \underline{Z}u, \tag{7}$$

where $\underline{X} = B_F$, $\underline{Z} = (B_R, Z)$, $u = (\beta_R, b)$, and $u \sim N(0, \Sigma_{\tau,\gamma})$ with

$$\Sigma_{\tau,\gamma} = \text{Cov}\,(u) = \begin{bmatrix} \Lambda_\tau & 0 \\ 0 & D_\gamma \end{bmatrix}.$$

Results of Twins Mortality Data Analysis

Table S1: Stratified comparisons of second and firstborn twins: adjusted ORs of perinatal death obtain from the Bayesian fit of the logistic additive mixed effects models

Variable	adjusted OR (95 % CI)			Variance of random intercepts		
	Bayesian-UNIF	Bayesian-HC	Bayesian-IG	Bayesian-UNIF	Bayesian-HC	Bayesian-IG
**Birth weight, heavier in %**[a]						
Heavier firstborn twin						
$\geq$ 25%	3.56 (2.38, 4.82)	3.42 (2.47, 4.70)	3.37 (2.30, 4.54)	3.9	3.5	2.1
15 to < 25%	2.05 (1.52, 2.60)	1.97 (1.58, 2.49)	1.94 (1.47, 2.41)	6.1	5.5	3.3
5 to < 15%	1.44 (1.16, 1.66)	1.39 (1.20, 1.62)	1.37 (1.12, 1.57)	4.9	4.4	2.6
Similar birth weight						
within ± 5%	1.32 (1.09, 1.45)	1.27 (1.13, 1.43)	1.25 (1.05, 1.38)	6.0	5.4	3.1
Heavier secondborn twin						
5 to < 15%	1.24 (0.94, 1.44)	1.19 (0.97, 1.40)	1.17 (0.90, 1.35)	5.2	4.7	2.7
15 to < 25%	0.95 (0.69, 1.23)	0.91 (0.71, 1.20)	0.89 (0.66, 1.16)	6.2	5.6	3.3
$\geq$ 25%	0.34 (0.24, 0.46)	0.33 (0.25, 0.45)	0.32 (0.23, 0.43)	3.6	3.3	2.0

[a] Birthweight difference in percentage comparing the heavier vs lighter twins.
